# A system-wide health sciences faculty mentor training program is associated with improved effective mentoring and institutional climate

**DOI:** 10.1017/cts.2021.883

**Published:** 2021-12-23

**Authors:** JoAnn Trejo, Deborah Wingard, Virginia Hazen, Alexandra Bortnick, Karen Van Hoesen, Angela Byars-Winston, Christine Pfund, Vivian Reznik

**Affiliations:** 1 Department of Pharmacology, School of Medicine, University of California, San Diego, La Jolla, CA, USA; 2 Health Sciences Office of Faculty Affairs, University of California, San Diego, La Jolla, CA, USA; 3 Herbert Wertheim School of Public Health and Human Longevity, University of California, San Diego, La Jolla, CA, USA; 4 Department of Emergency Medicine, School of Medicine, University of California, San Diego, La Jolla, CA, USA; 5 Department of Medicine, School of Medicine, University of Wisconsin-Madison, Madison, WI, USA; 6 Wisconsin Center for Education Research, University of Wisconsin-Madison, Madison, WI, USA; 7 Department of Pediatrics, School of Medicine, University of California, San Diego, La Jolla, CA, USA

**Keywords:** Behavior, clinical, diversity, inclusion, medicine, minority, racism, science

## Abstract

**Introduction:**

Mentorship is critical for faculty success, satisfaction, and engagement. However, many faculty, particularly underrepresented racial/ethnic (UR) faculty, lack access to high-quality mentoring. In an effort to improve mentoring for all faculty, we developed and implemented a formally structured faculty mentor training program (FMTP) across UC San Diego Health Sciences, which included institutional support, mentorship training, and department/division mentorship programs.

**Methods:**

FMTP impact was evaluated using three primary outcome variables: mentoring quality, mentoring behaviors, and institutional climate. Participants’ self-assessed mentoring competencies were measured using validated instruments.

**Results:**

A total of 391 (23%) of Health Sciences faculty participated in FMTP. Participation rate was higher for women than men (30% versus 17%) and highest for UR faculty (39%). FMTP was implemented in 16 of 19 departments. Self-reported mentoring improved for FMTP participants with mentoring quality (*p* = 0.009) and meeting mentees’ expectations (*p* = 0.01) continuing to improve for up to 2 years after training. However, participants were unsure if they were meeting UR mentees’ expectations. FMTP participants were significantly more satisfied with mentoring quality (*p* < 0.001) compared to non-participants, with the greatest increase in satisfaction reported by UR faculty (38–61%). UR faculty reported improved overall morale (51–61%) and a perception that the environment was supportive for UR faculty (48–70%).

**Conclusion:**

The implementation of a system-wide formal structured FMTP was associated with improved faculty satisfaction, quality of mentoring, and institutional climate, especially for UR faculty.

## Introduction

Mentorship has a critical role in career advancement and success for all faculty, including clinical, clinical translational, and basic science researchers in academic medicine and science [1–3]. Since the 1990s, there has been recognition for the need for effective mentoring programs leading to the creation of various formal and informal mentoring programs for faculty in academic medicine [1,4–6]. However, existing formal mentoring programs often lack evidence-based curriculum for mentorship education, centrally organized infrastructure and resources, uniform implementation across departments, and a cohesive mentoring community. Most mentorship programs are developed for faculty building research careers with little attention to mentoring clinical faculty and educators, a population most vulnerable to burnout [7,8]. In addition, historically underrepresented racial/ethnic (UR) faculty fail to receive high-quality mentoring [9]. The lack of high-quality effective mentoring can result in low self-efficacy, slowed career advancement, reduced research, scholarly productivity, and satisfaction [1,10–12].

High-quality mentorship not only improves individual success but also impacts the organization by improving faculty retention, satisfaction, and institutional engagement [4,7,13]. This is particularly relevant to UR faculty that often experience unwelcoming institutional cultures and a lack of sense of belonging [14,15]. Initiatives to optimize mentorship for all faculty promote inclusive excellence by fostering a positive and welcoming climate that values, includes, and supports all faculty. Of the formal system-wide mentorship programs established in academic medical institutions, most showed improvement in mentoring competencies and satisfaction, but to our knowledge none have evaluated the impact of formal mentorship programs on mentoring effectiveness or institutional climate including faculty morale and supportive environment, critical elements of inclusion [16–18].

In 2017, the University of California (UC) San Diego Health Sciences lacked an institutionalized, formally structured faculty mentorship program, similar to most Association of American Medical Colleges (AAMC) institutions. Faculty mentorship was limited in scope and lacked uniformity across all schools, departments, and divisions. There was no evidence-based mentorship training available for faculty, no process for implementing and evaluating mentoring effectiveness, and a lack of leadership engagement around mentoring in the departments. Mentorship was inaccessible and ineffective for the majority of junior faculty. In an effort to improve mentoring for all faculty, the UC San Diego Health Sciences Office of Faculty Affairs (OFA) embarked on a multi-year, multi-faceted approach to establish a system-wide formal faculty mentor training program (FMTP). The impact of UC San Diego FMTP was assessed using multiple outcome variables including mentoring quality, mentoring behaviors, and institutional climate.

UC San Diego Health Sciences is a research-intensive academic healthcare institution with 1723 faculty appointed in the School of Medicine (with 19 departments), the Skaggs School of Pharmacy and Pharmaceutical Sciences and the Herbert Wertheim School of Public Health and Human Longevity, founded in 2020. Approximately ∼60% are clinical and ∼40% are research faculty. Forty-four percent of the faculty are women and 9% are URs. The large diverse faculty appointed in various professional schools in Health Sciences at UC San Diego is typical of the 151 MD- and PhD-granting institutions in the AAMC consortium. Here, we describe steps for the successful development and implementation of a system-wide Health Sciences FMTP that required institutional support, centralized implementation of training curriculum and assessment, senior faculty engagement, and departmental leadership commitment. We provide data of FMTP impact on faculty mentoring skills, satisfaction with mentoring quality, mentoring behaviors and on institutional climate including faculty morale and supportive environment.

## Methods

### Development and Implementation of FMTP

Before initiation of FMTP, an online mentoring survey was developed and administered to all faculty in 2017 to assess their mentor training and experience. Seventy percent of the 525 faculty respondents (35% response rate) had served in a mentor role; only 22% had participated in a formal mentoring program, 75% believed that formal mentor training was important, and 83% believed formal training in mentoring would enhance their skills. These results suggested that Health Sciences faculty value mentorship education.

OFA sought leadership support to develop and fund a system-wide formal evidence-based, structured FMTP. The UC San Diego Chancellor and Vice-Chancellor of Health Sciences provided institutional funds with ancillary funds provided by the UC San Diego Altman Clinical Translational Research Institution to support FMTP for a 3-year pilot period beginning in 2017 with the goal of enhancing effective mentorship through evidence-based interventions and creating uniform quality mentoring across Health Sciences. The goal was to achieve a critical mass (∼30%) of faculty trained in effective mentorship, including senior faculty mentors, junior faculty mentees, and a cadre of Facilitator trainers to implement mentor training and sustain the program within 3 years. All Health Sciences faculty were eligible to participate.

### FMTP

**Fig. 1.**
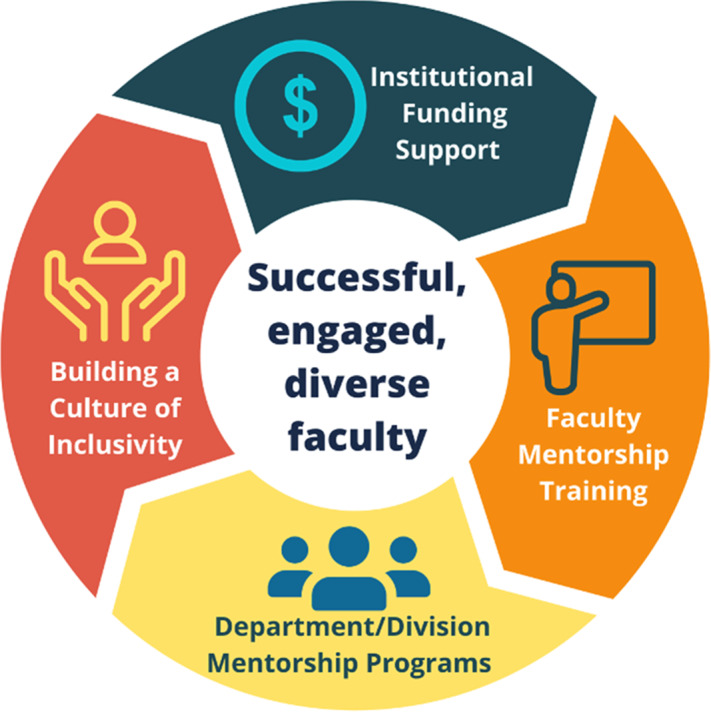
Four key elements of the UC San Diego Health Sciences Faculty Mentor Training Program (FMTP).

FMTP has four elements (Fig. 1). The first element is securing institutional support for the implementation of effective mentorship across Health Sciences. The Office of Faculty Affairs (OFA) recognized the need for a centralized, structured mentoring program for Health Sciences faculty and obtained funding from the institution for program personnel, training expenses, and evaluation (∼$70,000 per year). OFA also provides small grants to departments ($2000 each) and divisions ($500 each) for implementation of mentoring programs and to support other mentoring activities. Additionally, OFA sponsors senior faculty to participate in Facilitator training workshops to learn to implement mentor training (∼$8500 to train 5 faculty per year).

The second element of FMTP is the centralized mentorship training of senior and junior faculty to address the need for competency-based practice. Senior faculty attend an 8-hour face-to-face mentor training workshop, based upon the evidence-based curriculum, *Entering Mentoring* [19]. This curriculum covers key competencies including maintaining effective communication, aligning expectations, assessing understanding, fostering independence, building career self-efficacy [20], promoting work-life integration, and addressing equity and inclusion [2,21,22]. Master Facilitators from the Center for the Improvement of Mentored Experiences in Research (CIMER, www.cimerproject.org) led the training. Faculty become FMTP Certified Mentors after completing the 8-hour training, submitting evaluations, and writing a mentoring philosophy statement. These activities completed within a month provide time for reflection about the mentoring process and experience as an intentional mentor practitioner. Faculty receive Continuing Medical Education (CME) credits for participation.

Junior faculty receive mentee training in how to manage and maximize their mentoring relationships [23] by attending a 3-hour face-to-face Mentoring Up training workshop facilitated by Master Facilitators with the National Research Mentoring Network (NRMN) [24] and CIMER. This training is based upon the Mentoring Up curriculum which helps junior faculty develop the knowledge and skills to navigate their mentoring relationships and career progression proactively and effectively. Topics include maintaining effective communication, aligning expectations, achieving independence, and promoting professional development.

All faculty members engaged in mentor training receive an FMTP Toolkit designed and developed by OFA. The toolkit contains resources for advancing mentoring practices across each of the targeted competencies and a junior faculty Career Development Plan (CDP) focused on academic promotion. The toolkit “Preparing for Success” section includes guidance for establishing effective communication and trust, “Logistical Tools” section contains first and annual mentor meeting checklists, and the “Evidence-Based Tools & Activities” section includes mentoring compact and instructions on developing a mentoring philosophy.

Each year, OFA sponsors up to five senior faculty to participate in a 2-day CIMER conducted Facilitator Training [25–27]. Participants use the *Entering Mentoring* curricula to practice facilitating training components and to develop a plan for implementing the training at their institution. These trained Facilitators are expected to implement mentor training for faculty and help meet the growing need and demand for effective and culturally responsive mentorship training in their units and across Health Sciences.

The establishment of the department-specific mentoring programs is the third element of FMTP. Since Health Sciences is a heterogenous environment (e.g., various compositions of researchers and clinicians, rank, and culture of each department/division), FMTP is designed to allow individual departments/divisions to construct their own distinctive mentorship programs facilitated by the Department Chair or in large departments by a Division Chief. For each program, one to two department or division mentor directors (DMDs) are selected to implement their unique program. DMDs are FMTP certified faculty mentors who have direct knowledge of the culture in their units, the academic promotion process and policies, and professional development opportunities. DMDs coordinate mentor-mentee pairings/groupings, provide oversight, guidance, and support to department/division faculty and report annually to OFA. Creating a community of mentoring excellence is facilitated by OFA hosted quarterly meetings for DMDs or their delegates and faculty to share best practices, discuss challenges and potential solutions.

A fourth element of FMTP focuses on strategies that catalyze the creation of a more inclusive culture in Health Sciences. FMTP curriculum improves faculty mentoring skills and raises awareness of cultural differences of mentees from different race/ethnicity, gender, lesbian, gay, bisexual, transgender, and queer (LGBTQ) and other backgrounds. FMTP is one of several programs implemented by OFA with a specific purpose of fostering successful, engaged, and diverse faculty. The UC San Diego National Center of Leadership in Academic Medicine (NCLAM) and Hispanic Center of Excellence (HCOE) faculty development programs are two existing institutional programs with a focus on diversity and inclusion that have incorporated FMTP into their curriculum.

To increase the visibility of mentorship and mentoring excellence and to foster an inclusive culture of mentoring, OFA established the annual Health Sciences Mentoring Excellence Celebration. The celebration recognizes three Health Sciences faculty for their outstanding contributions to mentorship with an Excellence in Mentoring Award and features a nationally recognized expert on mentorship as keynote speaker.

### Data Collection and Analysis

The Kirkpatrick model was utilized to evaluate the impact of FMTP [28]. This four-step model measures reactions to the training program, learning, behaviors, and results. The first three steps were assessed through pre- and/or post-surveys of FMTP faculty workshop participants, while the fourth step was assessed through climate surveys of all Health Sciences faculty at UC San Diego. All surveys were administered online.

#### Reactions

During the last session of the FMTP training workshops, all participants were asked if the course learning objectives were met using a 5-point scale (1 = strongly disagree to 5 = strongly agree). In addition, participants were asked to rate the quality of content and presentation of the material on a 5-point scale (1 = poor, 2 = fair, 3 = average, 4 = good and 5 = excellent). Responses to the 5-point scales were summarized as a percent indicating agree/strongly agree or good/excellent.

#### Learning and behaviors

Skill levels for targeted mentoring competencies pre- and post-FMTP were self-assessed by junior and senior faculty training workshop participants using 1) a validated 7-point Mentoring Competency Assessment (MCA) scale [29], and 2) a validated 5-point Cultural Diversity Awareness (CDA) scale [30]. These surveys were administered online during the last session. A third follow-up survey was newly developed for this study and administered online to FMTP-trained senior faculty 6 months to 2 years after the training workshop. This follow-up survey included the same validated MCA scale as well as new non-validated questions measuring overall quality of mentoring provided, confidence in mentoring, mentoring behaviors adapted, and motivation for change. Responses to the 7-point scales were summarized both as means and percent.

#### Results

Three anonymous online surveys administered to all Health Sciences faculty were used to assess impact of FMTP. The first, a climate survey, was conducted in 2015. The second, a mentoring survey, was conducted in 2017 prior to the initiation of the FMTP. This survey helped inform the structure and scope of FMTP. The third, another climate survey, was conducted in late 2019 after the FMTP was well underway. Morale and assessment of a supportive environment for women or UR faculty were assessed on both climate surveys and compared over time for all respondents. Satisfaction with quality of mentoring received was assessed on the 2017 mentoring survey and the 2019 climate survey and compared over time for all respondents. In addition, satisfaction with quality of mentoring received and morale reported in 2019 by those who had participated in FMTP were compared to those who reported not participating in FMTP or programs that integrated FMTP including NCLAM and HCOE.

Morale was assessed by the question “Thinking about all aspects of my professional life at UCSD, my current morale is….” Responses were dichotomized as excellent or very good (1–2) versus fair or poor (3–4). Environment was assessed by “UCSD provides a supportive environment for…” each of several groups including, female faculty, male faculty, ethnic/racial minority faculty, and LGBTQ faculty. Responses were dichotomized as strongly or somewhat agree (1–2) versus somewhat or strongly disagree (3–4). Quality of mentoring received was assessed by agreement with the statement “I am satisfied with the quality of career mentoring I have received at UCSD” using a 7-point scale (1 = strongly agree to 7 = strongly disagree). Responses were dichotomized as 1–3 compared to 4–7.

Responses to the scales were summarized as means and percent. Differences in mean values for scales were tested by the Mann-Whitney U test. Differences in percent distribution of dichotomized scales were tested by Fisher’s exact test (two-tailed). The data were analyzed using R 4.0.2 (R Core Team, Vienna, Austria). Changes over time were not tested for significant differences, as there was overlap of respondents to the anonymous surveys which violates the requirement of independent samples. Since surveys were anonymous, there was no way to conduct a sub-analysis restricted to the same respondents at both time points.

### Ethical Approval

The UC San Diego Human Research Protections Program approved the mentoring study (Project #180614QI) and the climate surveys (Project #160540XX).

## Results

### Participation

#### Institutional support and FMTP funding

OFA supported the training of 391 faculty between 2017 and 2020. Sixteen departments and six Department of Medicine (DOM) divisions in the School of Medicine and the School of Pharmacy received funding for mentoring activities. As reported in annual DMD reports and at quarterly DMD meetings, funds were used to support mentoring retreats, mentoring steering committee meetings, invited speakers, mentor and grant writing training sessions and establishing an iShare website for mentors and mentees to exchange materials such as CDPs. Nine senior faculty mentors from seven departments and two divisions in DOM participated in CIMER facilitator training. These trained Facilitators are implementing mentor training of faculty, residents, fellows, and postdoctoral scholars and have conducted 13 mentor training sessions.

#### Faculty mentorship training

Approximately 23% (391/1723) of Health Sciences faculty participated in FMTP training between 2017 and 2020. Mentor training workshops for junior faculty totaled three hours and included 25–40 individuals each, while workshops for senior faculty totaled eight hours and included 30–35 individuals per session. As shown in Table 1, 57% of the participants were women and 15% were URs, which is higher than their representation among all faculty (44% women and 8.6% UR). Most of the FMTP participants were either assistant or full professors, similar to overall Health Sciences faculty. Approximately half of participants were in the clinical series, compared to 63% of the total clinical faculty, reflective of overall Health Sciences faculty demographics. The 57 UR included 45 Hispanic/LatinX, 8 African American/Black, 2 American Indian/Alaska Native, and 2 Filipino faculty members. Among senior faculty participants who had completed FMTP training at least 6 months earlier, approximately 50% (88/177) responded to a follow-up survey about mentoring confidence and behavior change (Table 1).

**Table 1.** Characteristics of faculty participants (n = 391) in the UC San Diego FMTP, 2017–2020

		Training workshop participants						Follow-up survey participants	
		Total		Junior faculty		Senior faculty		Senior faculty	
		*n*	%	*n*	%	*n*	%	*n*	%
Participants		391	100%	180	100%	211	100%	88	100%
Gender	Women	224	57%	115	64%	109	52%	52	59%
	Men	167	43%	65	36%	102	48%	36	41%
Ethnicity	UR	57	15%	36	20%	21	10%	8	9%
	Non-UR	334	85%	144	80%	190	90%	80	91%
Rank	Assistant	157	40%	155	86%	2	1%	1	1%
	Associate	79	20%	22	12%	57	27%	24	27%
	Full	148	38%	3	2%	145	69%	60	68%
	RTAD	7	2%	0	0%	7	3%	3	3%
Series	Research	188	48%	84	47%	104	49%	42	8%
	Clinical	196	50%	96	53%	100	47%	46	52%
	RTAD	7	2%	0	0%	7	3%	0	0%

FMTP = Faculty Mentor Training Program; UR = underrepresented racial/ethnic faculty; RTAD = retired with return to active duty, Junior Faculty = assistant professors, Senior Faculty = associate and full professors.

#### Department/division mentorship programs

To date, 16 of 19 departments in the School of Medicine have implemented FMTP and appointed one or two DMDs in the larger departments, with a total of thirty-four DMDs. FMTP was implemented in six divisions in the DOM because of its large size (>500 faculty). The School of Pharmacy has established a program with two DMDs, and the newly formed School of Public Health has committed to implementing a program. There have been eight DMD meetings to share progress and practices enhancing the culture of mentoring excellence.

#### Building a culture of inclusivity

All FMTP faculty participants were trained using evidence-based curriculum that addresses diversity and inclusion. The high percentage of women (57%) and UR faculty (15%) recruited to participate in FMTP not only enhances training in effective mentorship but also fosters connections among all faculty that may increase awareness and understanding of cultural differences. To build community, three Mentoring Excellence Celebrations were held, which included faculty Mentoring Excellence Awards and keynote lectures.

### Evaluation

The Kirkpatrick model was utilized to evaluate the impact of FMTP, using the four steps of reactions, learning, behaviors, and results [28].

#### Reactions

The first step in the Kirkpatrick model of program evaluation showed over 75% of the respondents rated the training content and presentation as excellent 82% (69/84) and 76% (64/84), respectively, all the rest rated content and presentation as good. A total of 72% of respondents strongly agreed that the course learning objectives were met, while 93% agreed or strongly agreed.

#### Learning

Self-assessed pre- and post-training surveys showed improvement in all mentoring competencies for both junior and senior faculty (data not shown) similar to published reports [2,31,32]. There was also significant improvement between completion of the mentor training workshop and a follow-up survey 6 months to 2 years later. Among the 73 senior faculty who responded at both times, overall quality of mentoring provided increased from 5.38 to 5.68 (*p* = 0.009) and meeting mentees’ expectations increased from 5.24 to 5.54 (*p* = 0.01). Four specific competencies improved significantly: establishing a relationship based on trust (*p* < 0.001), stimulating mentees’ creativity (*p* < 0.01), accurately estimating mentees’ level of scientific or clinical knowledge (*p* < 0.01), and taking into account the biases and prejudices you bring to your mentors/mentee relationship (*p* < 0.05). The two competencies that showed the most improvement for senior faculty after engagement in mentor training were aligning expectations and addressing diversity.

Among 88 senior faculty who responded to the follow-up survey 6 months to 2 years after the training workshop (Table 2), 88% reported being highly to extremely skilled in establishing a relationship on trust, 82% in building mentees’ confidence, while over 75% reported highly or extremely skilled for five other key competencies. The lowest skills levels were reported for three competencies related to issues of race, ethnicity, and bias.

**Table 2.** Self-assessed skill in mentoring competencies reported in 2020 among 88 senior level faculty who participated in the UC San Diego FMTP, 2017–2019

Mean^a^	Highly to extremely skilled		Competencies
	%^b^	(n/N)^c^	
**How skilled do you feel you are in each of the following mentoring areas?**			
5.91	88	(77/88)	Establish a relationship based on trust
5.62	82	(71/87)	Building mentees’ confidence
5.50	76	(67/88)	Aligning your expectations with your mentees
5.45	77	(67/87)	Understanding your impact as a role model
5.36	75	(66/88)	Stimulating your mentees’ creativity
5.32	77	(68/88)	Helping your mentees balance work with their personal life
5.27	75	(66/88)	Accurately estimating your mentees’ level of scientific or clinical knowledge
5.12	66	(58/88)	Taking into account the biases and prejudices you bring to your mentor-mentee relationship
4.63	49	(41/84)	Respectfully broaching the topic of race/ethnicity in my mentoring relationships
4.59	51	(43/85)	Intentionally creating opportunities for my mentees to bring up issues of race/ethnicity when they arise

FMTP = Faculty Mentor Training Program.

a Mean calculated on a 7-point scale, where 1 = not at all skilled and 7 = extremely skilled.

b Percent faculty responding 5–7 (highly skilled to extremely skilled).

c Total number responding 5–7 over total respondents (excluding don't know, not applicable and blank).

Among senior faculty who responded to the follow-up survey, 95% rated the overall quality of the mentoring they provided as high to very high (averaging 5.71 on the 7-point scale) and 90% felt they were meeting their menteesʼ expectations (averaging 5.59 on the 7-point scale). Senior faculty reported they were highly confident in their ability to mentor women (89%), UR (85%), and LGBTQ (81%) mentees effectively. Many of the participants, however, indicated they did not know if they were meeting their UR or LGBTQ mentees’ expectations or that the question was not applicable presumably because they did not have any such mentees (32% and 62%, respectively).

#### Behaviors

As the third step of the Kirkpatrick evaluation model, senior faculty were also asked about their mentoring behaviors in the follow-up survey. As shown in Table 3, 86% self-reported discussing work-life-integration with their mentees, 84% practicing active listening more regularly than before the training, and 70% discussed their expectations for the mentees’ independence. Over half of the mentors, 57% reported that they used Career Development Plans or Individual Development Plans (IDPs) with their mentees most of the time, and a third reported using the First Meeting Checklist, a resource in the FMTP Toolkit. Six months to two years after training, 98% of the senior faculty reported that participating in FMTP motivated their change in mentoring behavior.

**Table 3.** Mentoring behaviors adopted and motivation for change reported in 2020 by 88 senior level faculty who participated in the UC San Diego FMTP, 2017–2019

%^a^	(n/N)^b^	Mentoring behaviors and motivation
**Since the training, do you use the following with your mentees?** ^**c**^		
86	(76/88)	Discussing work-life integration with mentees
84	(74/88)	Practicing active listening more regularly than before the training
70	(62/88)	Articulating my expectations for independence
38	(33/88)	Developed a written mentoring philosophy
38	(33/88)	Discussing equity and inclusion research
6	(5/88)	Posted my approach to mentoring on my website/dept or program website
**Since the training, how often do you use the following with your mentees?** ^**d**^		
57	(49/86)	Career Development Plan (CDP)/Individual Development Plan (IDP)
53	(46/87)	Multiple approaches to assess understanding
34	(29/86)	First meeting checklist
21	(18/86)	Mentoring compacts
15	(13/85)	Communication Styles Inventory
**What motivated you to change your mentoring behaviors?** ^**e**^		
98	(86/88)	Faculty Mentor Training Program (FMTP)
72	(63/88)	Self-reflection
27	(24/88)	Scholarly research on mentoring
23	(20/88)	Feedback from mentees
9	(8/88)	Feedback from other faculty

FMTP = Faculty Mentor Training Program.

a Percent faculty responding as indicated.

b Total number responding as indicated over total respondents (excluding don't know, not applicable and blank).

c Responding “yes.”

d Responding “most of the time.”

e Selecting given motivation.

#### Results

As the fourth step in the Kirkpatrick evaluation model, quality of mentoring received, morale, and support for women faculty and UR faculty was assessed by surveying Health Sciences faculty before and after the establishment of the FMTP program. The first climate survey, prior to the initiation of the FMTP, was conducted in 2015 and had 631 respondents for a response rate of 42%. The mentoring survey, also prior to the FMTP, was conducted in 2017 and had 526 respondents for a response rate of 35%. The second climate survey was conducted in late 2019 after the FMTP was well underway and had 902 respondents for a response rate of 50%.

**Fig. 2.**
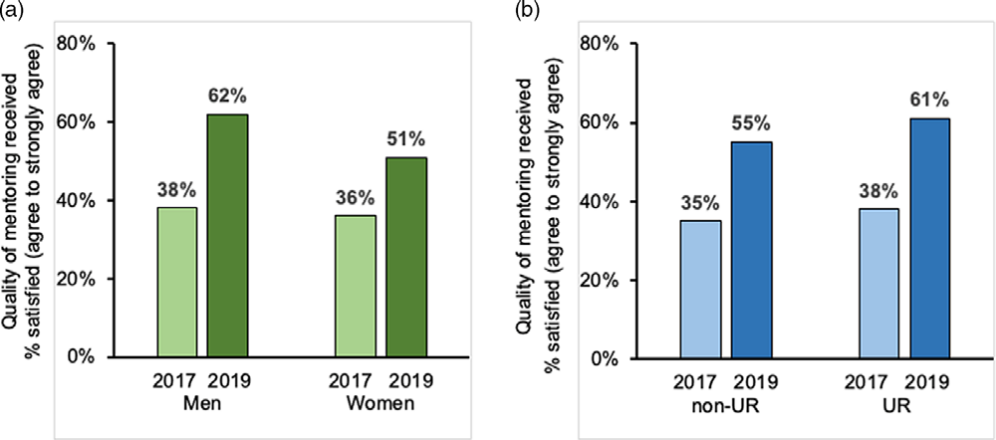
Satisfaction with quality of mentoring received at UC San Diego reported in 2017 and 2019 climate surveys of Health Sciences junior faculty by gender and underrepresented (UR) racial / ethnic backgrounds. a: Satisfaction with quality of mentoring received reported by men (25/66) and women (34/95) junior faculty in 2017 (*light green bars*) compared to men (56/90) and women (67/132) in 2019 (*dark green bars*). b: Satisfaction with quality of mentoring received reported by non-UR (52/147) and UR (8/21) junior faculty in 2017 (*light blue bars*) compared to non-UR (115/211) and UR (17/28) junior faculty in 2019 (*dark blue bars*).

In early 2017, 32% of all faculty respondents reported that they were satisfied with the quality of mentoring received at UC San Diego; satisfaction increased to 46% by late 2019. Satisfaction with quality of mentoring received among junior faculty (assistant professors) increased from 36–55% between 2017 and 2019. As shown in Fig. 2a and 2b, among junior faculty the greatest increase in satisfaction with quality of mentoring received was reported by men (38–62%) and UR faculty (38–61%).

**Fig. 3.**
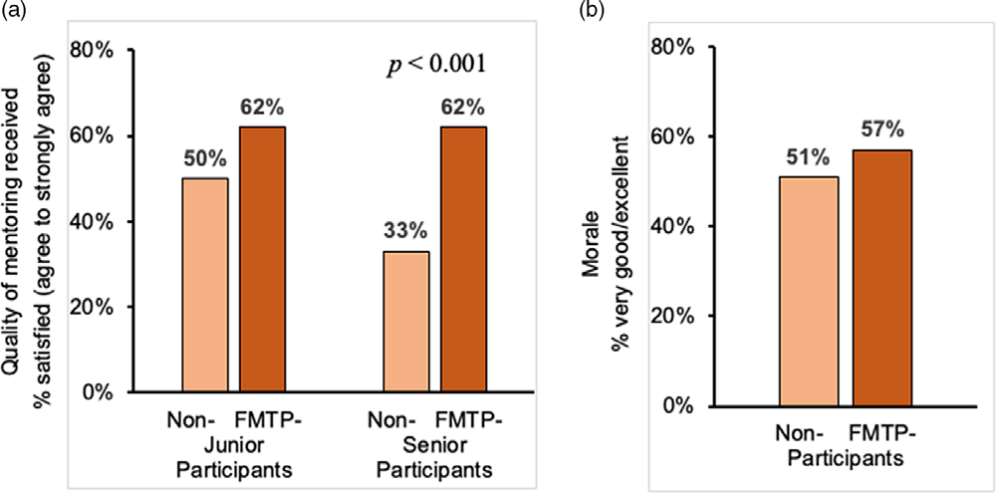
Satisfaction with quality of mentoring received and morale reported in a 2019 climate survey of all UC San Diego Health Sciences faculty who participated in FMTP compared to non-participants. a: Satisfaction with quality of mentoring received at UC San Diego reported by junior (40/65) and senior (88/142) FMTP faculty participants (*dark orange bars*) compared to junior (66/133) and senior (115/351) non-participants (*light orange bars*). b: Morale reported as very good to excellent by all FMTP participants (123/216) (*dark orange bars*) compared to non-participants (272/532) (*light orange bars*).

**Fig. 4.**
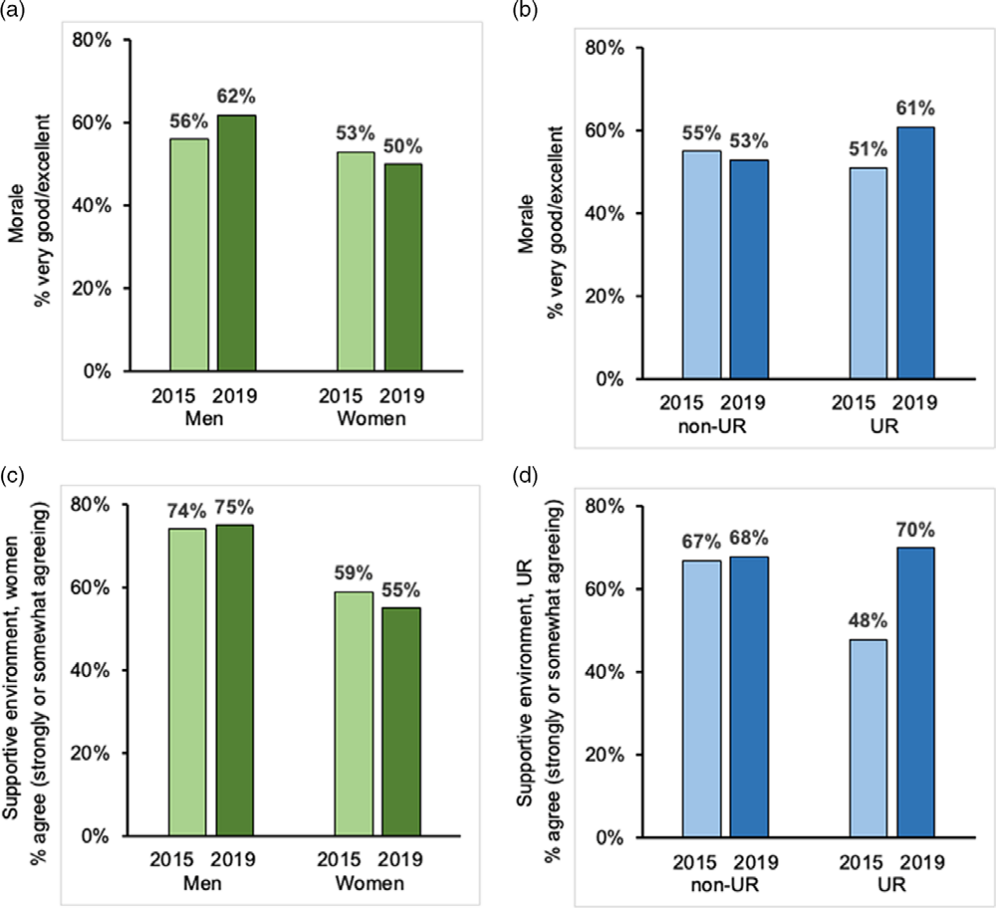
Morale and supportive environment reported in climate surveys of UC San Diego Health Sciences faculty in 2015 and 2019 by gender and underrepresented (UR) racial / ethnic backgrounds. a: Morale reported as very good to excellent by men (183/324) and women (155/295) faculty in 2015 (*light green bars*) compared to men (258/419) and women (204/411) faculty in 2019 (*dark green bars*). b: Morale reported as very good to excellent by non-UR (315/575) compared to UR (27/53) faculty in 2015 (*light blue bars*) compared to non-UR (442/833) and UR (42/69) faculty in 2019 (*dark blue bars*). c: Supportive environment for women faculty reported by men (241/325) and women (173/294) faculty in 2015 (*light green bars*) compared to men (314/419) and women (224/411) faculty in 2019 (*dark green bars*). d: Supportive environment for UR faculty reported by non-UR (385/574) and UR (25/52) faculty in 2015 (*light blue bars*) compared to non-UR (565/833) and UR (48/69) faculty in 2019 (*dark blue bars*).

A total of 216 faculty responding to the 2019 climate survey indicated that they had participated in the FMTP, while 532 faculty reported they had not participated in FMTP. Satisfaction with the quality of mentoring received was higher for junior faculty FMTP participants compared to non-participants, with a significance difference observed among senior faculty satisfaction with their quality of mentoring (62% vs 33%, *p* < 0.001) (Fig. 3a). FMTP participants were also more likely to report receiving adequate mentoring on academic advancement (74% vs 64%, *p* = 0.009) and career development (69% vs 52%, *p* < 0.001). As also shown in Fig. 3b, FMTP participants were more likely than non-participants to report their overall morale as very good to excellent (57% vs 51%). Between the 2015 and 2019 climate surveys, faculty morale reported as very good to excellent showed only a modest changed for men (56% vs. 62%) and women (53% vs. 50%) (Fig. 4a). However, morale markedly improved from 51–61% for UR faculty compared to non-UR faculty over that time (Fig. 4b). Women were less likely than men to agree that the environment was supportive for women, and this did not change over time (Fig. 4c). UR faculty were less likely to agree that the institution provided a supportive environment for UR faculty in 2015 compared to non-UR faculty. However, their agreement markedly increased from 48–70% between 2015 and 2019 (Fig. 4d).

## Discussion

A culture of mentoring excellence improves faculty satisfaction, engagement, and retention in academic medicine and science. However, there is a lack of access to and availability of high-quality effective mentoring for all faculty, especially for UR faculty. To address this need, we developed and implemented a Health Sciences formal structured FMTP with evidence-based curriculum, departmental-level mentorship, and comprehensive mentorship evaluation, and to date, we have trained 23% of our faculty. FMTP implementation is associated with faculty engagement, improved UR faculty morale, and inclusion as measured by faculty’s perceptions of their environment being supportive for women and UR in the institutional climate survey.

FMTP provided faculty with evidence-based, mentorship education. Prior studies have shown that faculty mentoring quality and competencies improved after participation in mentorship education [2,16]. This report demonstrates for the first time that these improvements persist over time. Senior faculty participants reported a significant improvement in the quality of their mentoring and their ability to meet their mentees’ expectations. At the time of follow-up, ranging from 6 months to 2 years, 98% of faculty reported that FMTP training motivated them to change their mentoring behaviors. Mentoring behaviors included discussion of relevant topics for junior faculty (e.g., work-life integration, establishing independence) and effective communication processes like active listening. Weger et al. showed that participants who received active listening responses felt more understood than those who received only advice or simple acknowledgments [33]. Creating an environment where junior faculty feel like they are genuinely heard and understood by their faculty mentors is especially important during the COVID-19 pandemic that has resulted in increased stress and uncertainty in personal and work lives.

Seventy-two percent of trained faculty reported self-reflection as a motivator for changing their mentoring behaviors. Research on behavior change, such as the transtheoretical model of change popularized by Prochaska and DiClimente, illustrates the impact of writing down intended behaviors as a precursor to actually engaging in those behaviors [34]. While other studies have examined the long-term impact of faculty mentoring programs on satisfaction and productivity [17,35,36], the impact of faculty mentor training on mentors’ behavior or motivation for change in behavior has not been well studied [37]. Our findings suggest that having faculty create mentoring philosophy statements as part of their mentorship education is valuable to include in future faculty mentoring programs.

FMTP implementation was positively associated with increased satisfaction with quality of mentorship received by junior faculty. From 2017–2019, satisfaction with mentoring increased by 14% for all Health Sciences faculty, and nearly half of the faculty were satisfied with the mentoring received. We found a sizeable program effect for FMTP; participants reported considerably greater satisfaction with their mentoring and received more mentoring for academic and career advancement than non-FMTP participants. A higher percentage of FMTP participants rated the institutional climate more favorably than did non-participants. Our findings are significant because previous studies reported that less than half of academic medical faculty receive formal mentoring and those who are mentored are more satisfied and less likely to consider leaving their institution [4,7,13].

FMTP participation had the greatest impact on UR faculty overall. UR faculty reported the greatest increases in satisfaction with mentoring quality, morale/institutional climate, and the perception of a supportive institutional environment for UR faculty after FMTP implementation. In previous studies, UR faculty have reported lower perception of inclusion and less satisfaction with the academic diversity climate, networking, and intention to remain at their current institution [14,15]. While several mentoring programs for UR faculty demonstrated improvements in scholar productivity, none examined UR faculty satisfaction with quality of mentoring or their perceptions of the institutional climate [31,38,39]. Professional development programs like NCLAM and HCOE can provide a community of practice and networks for junior faculty to thrive; our data suggest the added benefit of FMTP integrated into established faculty development programs enhances junior faculty engagement. Because many UR FMTP participants were also in NCLAM and HCOE, we acknowledge that implementation of the FMTP in combination with UR faculty development programs at UC San Diego likely has a positive, additive impact on morale and perceptions of the institutional inclusivity.

Mentoring excellence includes developing mentors who can effectively engage with and develop the talent of all individuals, particularly UR mentees [40]. Our senior faculty reported high confidence in their ability to mentor women, UR, and LGBTQ mentees effectively; however, they reported not knowing if they were meeting the expectations of UR and LGBTQ mentees. An awareness of cultural differences is an essential first step in encouraging faculty to promote UR inclusion and belonging [41–43]. However, raising awareness of cultural diversity without a skill set to effectively address cultural dynamics that may arise in their mentoring relationships can compromise mentoring effectiveness. Byars-Winston et al. designed the culturally aware mentoring (CAM) program to improve the confidence and capacity of faculty mentors to be culturally responsive. CAM is designed to be incorporated into existing mentoring programs following foundational mentorship education. Evaluation of CAM participants immediate to post-training and 18–24 months later showed that CAM participation enhances faculty’s capacity to recognize and respond to cultural differences within their mentoring relationships [40,41]. In the present age of racial reckoning, this is a critical professional development need to address racism inherent in academic medicine. UC San Diego Health Sciences plans to engage faculty in CAM training as an addition to our FMTP program towards improving mentoring excellence.

### Limitations

The improvements to climate observed in this study may not be directly related to the programs described but may have occurred for other reasons or by chance. However, the improvement also being reported by UR faculty and feedback from individual faculty members support a causal interpretation. The present study of a single institution may not apply to other schools; however, this study describes a comprehensive institution-wide intervention that can be used as a model and strongly suggests the need to address diversity and inclusion in academic medicine. Results may reflect self-selection of participants and social desirability bias in responding to the surveys. However, variations by type of participant (men versus women, UR versus non-UR) should be less affected by these issues.

## Conclusion

Implementation of a system-wide formal structured mentoring program was associated with improved faculty engagement, satisfaction, and quality of mentoring across Health Sciences. FMTP improved mentoring competencies for all faculty and motivated faculty to change their mentoring behavior over time. FMTP participation was associated with improved faculty morale and institutional climate. UR faculty showed the greatest improvement in satisfaction with the quality of mentoring and overall morale. Senior faculty reported not knowing whether they were meeting UR mentee expectations. Interventions such as CAM aimed at raising awareness and skill development of senior faculty mentors to recognize and respond to cultural differences within their mentoring relationships should be integrated into new and existing faculty mentoring programs to enhance inclusive excellence.
